# Life after 45 and before 60: the *Retrovirology *Prize

**DOI:** 10.1186/1742-4690-2-26

**Published:** 2005-04-15

**Authors:** Kuan-Teh Jeang

**Affiliations:** 1 the National Institutes of Health, Bethesda, MD, USA

## Abstract

*Retrovirology *announces an annual prize to recognize one outstanding mid-career retrovirologist.

## The silent majority

In his US presidential campaigns, Richard Nixon famously popularized the concept of a "silent majority". Fundamentally what Nixon meant was that the stalwart contributors in any given population tend to be a faceless and non-vocal "majority". Applying Nixon's term to science, where is our "silent majority"?

I recently returned from a large international meeting where numerous awards were presented to wunderkinds (i.e. Young Investigator Awards) and senior statesmen/stateswomen of science (i.e. Lifetime Achievement Awards). Curiously, I found subjectively that the majority of the best work was being presented by a glum-looking bunch of scientists traversing "midlife crises" (i.e. individuals who uncannily resemble me!). It struck me that arguably the most productive denizens of science are those in the 45 to 60 age group (who are no longer young investigators and not yet competitive for lifetime achievements); and they are our equivalent of Nixon's "silent majority".

## The *Retrovirology *Prize

To my knowledge, there is no significant scientific prize that is age-restricted to mid-career scientists (i.e. between the ages of 45 to 60). Although mid-life scientists do and have competed ably for numerous non-age restricted awards, a unique niche is fulfilled by a prize that specifically recognizes this increasingly large and productive age group. Moreover, I am not aware of a prize limited to retrovirologists. Beginning this year, *Retrovirology *will recognize annually through the *Retrovirology *Prize one 45- to 60- year-old retrovirologist.

## The selection process

This editorial kicks-off the 2005 call for nominations for the *Retrovirology *Prize. We envisage an approximate alternation of the Prize yearly between a non-HIV retrovirologist (2005 and odd years) and an HIV retrovirologist (2006 and even years). There can be some discretion on this criterion exercised from time-to-time by the selection committee. A nomination includes a statement (1000 words or less) of significant contributions to retrovirology research and a curriculum vitae of the nominee. The selection committee is composed of the Editors (currently, M. Benkirane, B. Berkhout, M. Fujii, K.T. Jeang, M. Lairmore, A. Lever, and M. Wainberg) of *Retrovirology*. All nominations to the selection committee must come through an Editorial Board member of *Retrovirology*. Candidates may self-nominate, but they must ask a *Retrovirology *Editorial board member to communicate their nominations to the selection committee. A list of Editorial Board members can be found at the *Retrovirology *website www.retrovirology.com. All *Retrovirology *Editors and Editorial Board members are eligible for nomination with the exception of the Editor-in-Chief who will administer the final selection decision. For 2005, all nominations must be received by June 1. The *Retrovirology *Prize consists of an attractive trophy, a cash award, and a biographical article published in *Retrovirology *about the winner and his or her scientific contributions to retrovirology.

